# Curcumin and Selenium Synergistically Alleviate Oxidative Stress in IPEC-J2 Cells and ICR Mice

**DOI:** 10.3390/biology14091117

**Published:** 2025-08-23

**Authors:** Yu Zheng, Jiali Liu, Junxin Li, Bohan Zheng, Qinjin Li, Xiaohong Huang, Zhaoyan Lin

**Affiliations:** 1 College of Animal Science, Fujian Agriculture and Forestry University, Fuzhou 350002, China; zy931891704@163.com (Y.Z.); 2021720930@yangtzeu.edu.cn (J.L.); 18059732920@163.com (J.L.); zhengbohan@fafu.edu.cn (B.Z.); liqinjin0809@163.com (Q.L.); 2 University Key Laboratory for Integrated Chinese Traditional and Western Veterinary Medicine and Animal Healthcare in Fujian Province/Fujian Key Laboratory of Traditional Chinese Veterinary Medicine and Animal Health, Fujian Agriculture and Forestry University, Fuzhou 350002, China

**Keywords:** curcumin, selenium, IPEC-J2, oxidative stress, gut microbiota

## Abstract

Oxidative stress is closely associated with diarrhea in piglets, and alleviating intestinal oxidative stress may emerge as an effective strategy for porcine diarrhea. Curcumin (Cur) and selenium (Se) are both well recognized for their potent antioxidant effects. This study established in vitro (IPEC-J2 cells) and in vivo (ICR mice) intestinal oxidative stress models to investigate the effects and mechanisms of Cur combined with Se in alleviating oxidative stress. The results showed that Cur and Se exhibited synergistic antioxidant effects in vitro, outperforming individual treatments. Additionally, pretreatment with Cur and Se significantly attenuated dextran sulfate sodium (DSS)-induced colitis in ICR mice and improved serum antioxidant indices. Specifically, the mRNA levels of Nrf2, HO-1, and NQO-1 were upregulated, while the mRNA levels of NF-κB, IL-1β, and TNF-α were downregulated in colonic tissues. Finally, the 16S rRNA sequencing showed that DSS reduced alpha diversity and increased Simpson indices, while Cur and Se restored diversity indices and normalized beta diversity. To summarize, Cur and Se synergistically alleviate oxidative stress in IPEC-J2 cells and ICR mice, demonstrating therapeutic potential for intestinal disorders in pigs.

## Simple Summary

Piglet diarrhea represents a significant challenge in swine production, with oxidative stress implicated in its pathogenesis. Curcumin and selenium, as natural antioxidants, have the potential to alleviate piglet diarrhea. This study aimed to investigate the synergistic antioxidant effects of curcumin and selenium both in vitro and in vivo, as well as the underlying mechanisms. The results demonstrated that the combination of these two compounds significantly alleviated oxidative stress in porcine intestinal epithelial cells induced by hydrogen peroxide and ameliorate colitis in mice induced by dextran sulfate sodium, with better effects than monotherapy. Additionally, the antioxidant effects of curcumin and selenium were associated with the nuclear factor E2-related factor 2 and nuclear factor κ-B signaling pathways. The findings of this study will facilitate further research and application of curcumin and selenium in the treatment of piglet diarrhea.

## 1. Introduction

Oxidative stress is a state of excessive free radical production or insufficient scavenging capacity in the body, resulting in an imbalance between the oxidative and antioxidant systems [1]. Reactive oxygen species (ROS) are a class of highly chemically reactive oxygen-containing molecules, which are implicated in diverse physiological functions in organisms [2]. Abnormally elevated levels of ROS can trigger a cascade of adverse reactions, including disturbances in energy metabolism and alterations in protein structure, thereby affecting the function of cells, tissues, organs, and even systems [3].

Oxidative stress is closely associated with diarrhea in piglets [4], and the intestine is a major organ of oxygen radical attack [5]. Some studies have shown that oxidative stress leads to decreased expression or impaired function of nuclear factor E2-related factor 2 (Nrf2) and heme oxygenase-1 (HO-1) in intestinal epithelial cells, which disrupts the integrity of the intestinal barrier [6,7,8]. Gut microbiota is critical for livestock and poultry gut health, and studies have shown that the gut microbiota contributes to the alleviation of oxidative stress via its own metabolites or by producing secondary metabolites [9,10].

Curcumin (Cur) is a polyphenolic compound extracted from turmeric, which has been identified to possess significant antioxidant [11,12,13], anti-inflammatory [14,15], and anti-tumor [16] properties. Although the pharmacological effects of Cur are widely recognized, it suffers from low solubility, low stability, and low bioavailability in clinical applications [17,18]. Therefore, strategies to enhance the bioavailability of Cur have been proposed, such as combination therapy [19,20] and the use of nanocarriers [21].

Selenium (Se) is a trace element, which is involved in multiple biological functions such as antioxidant [22,23], anti-inflammatory [24], anti-tumor [25], etc. The antioxidant mechanism of Se is closely related to a Se-containing antioxidant enzyme: glutathione peroxidase 4 (GPX4) [26]. In addition, Se has a great effect in maintaining the intestinal microbial balance. Yeast selenium supplementation decreased the microorganism abundance of facultatively anaerobic and potentially pathogenic phenotypes of laying hens and regulated the microbiota composition of the cecum, which resulted in a healthier gut [27].

The combination application of drugs has multiple advantages. Through the synergistic effect between drugs, the dosage of each drug can be effectively reduced, thereby reducing the risk of drug residue and toxicity [28,29]. Several studies have shown that combining different antioxidants can enhance antioxidant efficacy, reduce individual doses to minimize side effects, and delay resistance development [30,31,32].

The Nrf2/HO-1/NAD(P)H dehydrogenase [quinone]-1 (NQO-1) signaling pathway plays a key role in maintaining intracellular redox homeostasis, defending against external damage, and regulating a variety of physiological and pathological processes [33,34]. Activation of the Nrf2/HO-1/NQO-1 pathway can effectively alleviate piglet diarrhea [35]. Additionally, the intestinal inflammatory response often accompanies diarrhea, with nuclear factor κ-B (NF-κB) signaling pathway activation being a key factor in the inflammatory process [36]. Inhibition of the NF-κB pathway can effectively mitigate intestinal inflammation [37,38].

In this study, the synergistic antioxidant properties of Cur and Se were confirmed in vitro and in vivo using IPEC-J2 cells and ICR mice, and the potential mechanisms underlying this synergistic antioxidant action were also explored. The results are expected to facilitate the future application of Cur and Se in antioxidant strategies for swine feeding.

## 2. Materials and Methods

### 2.1. Cell Maintenance

The intestinal porcine epithelial cell line (IPEC-J2, passage 3, Beina Biotechnology Co., Beijing, China) was cultured in DME/F-12 medium (HyClone, Logan, UT, USA) with 10% fetal bovine serum (Thermo Scientific, Waltham, MA, USA) and 1% penicillin/streptomycin (Solarbio Life Sciences, Beijing, China). Cells were maintained at 37 °C in a humidified incubator with 5% CO_2_.

### 2.2. Drug Treatment

Cur (molecular formula: C_21_H_20_O_6_, molecular weight: 368.38) was sourced from MedChemExpress Inc. (HY-N0005, Monmouth Junction, NJ, USA) and dissolved in anhydrous ethanol. Sodium selenite (chemical formula: Na_2_SeO_3_, molecular weight: 172.938, solubility in water: ≥200 mg/mL) was sourced from Sigma-Aldrich, Inc. (214485, St. Louis, MO, USA) and dissolved in double distilled water (ddH_2_O). IPEC-J2 cells were subjected to treatment with Cur, Se, or Cur plus Se for 24 h, after which H_2_O_2_ was added to the culture medium and lasted for 2 h.

### 2.3. Animals and Treatments

The animal study was authorized by the Animal Ethics Committee of Fujian Agriculture and Forestry University (authorization code: PZCASFAFU24052), according to the guidelines for Laboratory Animal Use and Care from the Chinese Center for Disease Control and Prevention and the Rules for Medical Laboratory Animals (1998) from the Chinese Ministry of Health.

A total of 70 ICR mice (male, SPF, 6 weeks of age, Wu’s Animal Health Products Co., Ltd., Fuzhou, China) were randomly divided into seven groups (Table 1). Mice were orally gavaged with drugs for 14 d, followed by the induction of intestinal oxidative stress via the addition of 3% dextran sulfate sodium (DSS) in drinking water for another 7 d.

During the experiment, the body weight of the mice was measured daily. The weight loss rate, fecal shape, and hematochezia (bloody stool) were scored according to the criteria provided in Supplementary Table S1. Finally, the disease activity index (DAI) for each group of mice was calculated using the formula: DAI = (weight loss score + stool consistency score + hematochezia score)/3. At the end of the animal experiments, the blood, feces, and colonic tissues of the mice were collected for subsequent experiments.

### 2.4. Cell Viability Assay

Cell viability was detected using Cell Counting Kit 8 (CCK-8, glpbio, Montclair, NJ, USA). Briefly, IPEC-J2 cells (1 × 10^4^ cells/mL) were seeded in 96-well plates, treated with different doses of Cur or Se for 24 h, or treated with different doses of H_2_O_2_ for 2 h. The CCK-8 reagent was subsequently added into the wells and cells were incubated for 30 min. The OD_450 nm_ value of each well was measured with a microplate reader (22202-SANGMSMA1, Molecular Devices, Shanghai, China). The mean optical density (OD, absorbance) of five wells in the indicated groups was used to calculate the percentage of cell viability as follows: percentage of cell viability = A_treatment_/A_control_ × 100% (where A = absorbance). The average absorbance of the control group was set to 100%. The experiments were carried out in triplicate.

### 2.5. Antioxidant Capacity Assays

The total antioxidant capacity of cells was measured using the Total Antioxidant Capacity Assay Kit employing the ferric reducing ability of plasma (FRAP) method (Beyotime Biotechnology Inc., Shanghai, China). Additionally, the superoxide dismutase (SOD) activity was measured using the SOD Assay Kit (Nanjing Jiancheng Bioengineering Inc., Nanjing, China), the malondialdehyde (MDA) levels were detected using an MDA Assay Kit (TBA method, Nanjing Jiancheng Bioengineering Inc., Nanjing, China), the catalase (CAT) and the glutathione (GSH) levels were detected using a CAT Assay Kit and GSH Assay Kit (Beyotime Biotechnology Inc., Shanghai, China), respectively. Briefly, IPEC-J2 cells were treated with drugs, then cells were collected and homogenized, and the supernatant or the mouse serum was collected into 96-well plates followed by the addition of appropriate reagents. The results were quantified with a microplate reader (22202-SANGMSMA1, Molecular Devices, Shanghai, China).

### 2.6. ROS Assay

ROS levels in IPEC-J2 cells were quantified using a ROS Detection Kit (Beyotime Biotechnology Inc., Shanghai, China). Cells were treated with drugs, then the DCFH-DA probe was diluted with serum-free culture, added to the plates, and incubated at 37 °C for 20 min. The images were acquired using a fluorescence microscope (IRX50, Sunny Optical Technology Inc., Yuyao, China), and the fluorescence intensity was measured using ImageJ (Version 1.52i, National Institutes of Health, USA) software. The experiments were carried out in triplicate.

### 2.7. Mitochondrial Membrane Potential (MMP) Assay

IPEC-J2 cells (1 × 10^6^ cells/mL) were seeded in 6-well plates for drug treatments, and the MMP was detected using an MMP Assay Kit with JC-1 (Beyotime Biotechnology Inc., China), the images were acquired using a fluorescence microscope (IRX50, Sunny Optical Technology Inc., Yuyao, China), and the fluorescence intensity was measured with ImageJ (Version 1.52i, National Institutes of Health, USA) software. The experiments were carried out in triplicate.

### 2.8. Transmission Electron Microscope (TEM) Scanning Assay

Briefly, IPEC-J2 cells were treated with drugs, then cells were harvested and fixed using 2.5% glutaraldehyde, followed by dehydration through a graded series of ethanol. The samples were then dried. Ultimately, the morphology of the cells was observed using TEM (HT7800, Hitachi Group, Tokyo, Japan). The experiments were carried out in triplicate and nine photographs were acquired for each group.

### 2.9. RNA Isolation and RT-qPCR Assay

The mouse colon tissue RNA was extracted using an AG RNAex Pro Kit (AG21101, Acray Biotech, Changsha, China), RNA concentration was determined by a 260/280 nm absorbance ratio, total RNA was reverse transcribed to cDNA using an Evo M-MLV Reverse Transcription Kit (AG11705, AcrayBio, Changsha, China), and a fluorescence quantitative analyzer (CFX96, BOLE Life Medical Products Co., Ltd., Shanghai, China) was used to detect the expression of *Nrf2*, *HO*-1, *NQO*-1, *NF-κB*, *IL*-1*β*, and *TNF-α* genes. The mRNA expression levels were calculated using the 2^ΔΔCt^ method. The primers were synthesized by Bioengineering Co. (Shanghai, China) and the primer sequences are shown in Table 2. The experiment was repeated six times (n = 6).

### 2.10. H&E Staining and Immunohistochemical (IHC) Assay

The mice colon tissue samples were firstly fixed in 4% formaldehyde solution (pH 7.4) at 4 °C for 48 h. Following dehydration, the tissues were embedded in paraffin wax and sectioned at a thickness of 4 μm. The paraffin sections were dewaxed to water, then subjected to H&E staining. For IHC assays, the slides were subjected to deparaffinization and antigen retrieval, then incubated with ZO-1 primary antibody (BS46078, Bioworld Biotech Inc., Nanjing, China, 1:300) at 4 °C for 16 h. Incubation was then performed with biotinylated secondary antibodies at 37 °C for 1 h. Then diaminobenzidine staining was performed and sections were counterstained with hematoxylin. The experiments were carried out in triplicate and six photographs were taken for each group.

### 2.11. 16s rRNA Gene Sequencing

Microbial DNA was extracted from mouse feces samples using the E.Z.N.A.^®^ Soil DNA Kit (Omega Bio-tek, Norcross, GA, USA). The V1–V9 region of the bacterial 16s ribosomal RNA gene was amplified by PCR using primers 27F 5′-AGRGTTYGATYMTGGCTCAG-3′ and 1492R 5′-RGYTACCTTGTTACGACTT-3′. Amplicons were excised from 2% agarose gels and purified using the AxyPrep DNA Gel Extraction Kit (Axygen Biosciences, Union City, CA, USA). SMRTbell libraries were prepared from the amplified DNA by blunt-ligation with SMRTbell Prep Kit 3.0 (Pacific Biosciences, PN: 102-182-700). Purified SMRTbell libraries from the pooled and barcoded samples were sequenced on a single PacBio Sequel IIe cell. All amplicon sequencing was performed by Shanghai Biozeron Biotechnology Co. Ltd. (China). Operational taxonomic units (OTUs) were clustered at a similarity threshold of 98.65% using UPARSE (version 10, http://www.drive5.com/uparse/ (accessed on 17 March 2025)). The rarefaction analysis based on Mothur (v.1.21.1) was conducted to reveal the alpha diversity indices, and the beta diversity analysis was performed using Euclidean distance matrix to principal component analysis (PCA) and non-metric multidimensional scaling (NMDS) with the *vegan* community ecology package, R-forge (https://r-forge.r-project.org/ (accessed on 20 March 2025)). The experiment was conducted in quadruplicate (n = 4). The raw sequencing data have been deposited in the NCBI SRA database (accession number: PRJNA1291475).

### 2.12. Statistical Analysis

The interaction between Cur and Se was assessed via calculation of the combination index (CI) using Compusyn software (version 1.0, Paramus, NJ, USA). CI values of less than 1 indicated synergism, values greater than 1 indicated antagonism, and a value of 1 indicated an additive effect.

Statistical analysis was performed using GraphPad Prism software (version 9.0, GraphPad Software Inc., San Diego, CA, USA) and SPSS software (version 25.0, IBM Inc., Armonk, NY, USA) with one-way ANOVA for multiple intergroup comparisons followed by a Tukey test for multiple comparisons, and the results of the analyses were presented as mean ± standard deviation (mean ± SD). A *p* value of 0.05 or less indicated statistical significance.

## 3. Results

### 3.1. Cur and Se Exert Synergistic Antioxidant Effects in IPEC-J2 Cells

**Figure 1:**
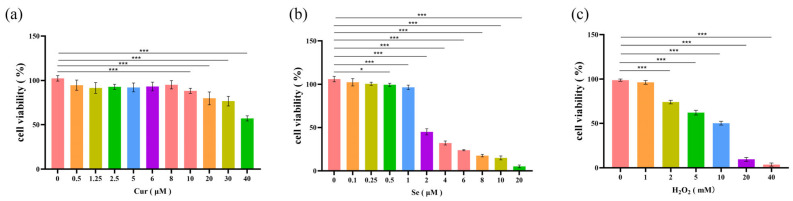
The drug application concentrations were determined by cell viability assays. IPEC-J2 cells were treated with different doses of Cur of Se for 24 (**a**,**b**) or treated with different doses of H_2_O_2_ for 2 h (**c**). Then the cell viability was detected by Cell Counting Kit 8. The average absorbance of the control group was set to 100%. The experiments were carried out in triplicate (n = 3). Abbreviation: Cur, Curcumin. Se, Selenium. *, *p* < 0.05; ***, *p* < 0.001.

In order to determine the appropriate drug concentrations, cell viability was assessed following the application of Cur, Se, or H_2_O_2_, and the results indicated that, when the concentration of Cur was below 20 μM and that of Se was below 2 μM, the viability of IPEC-J2 cells did not significantly decrease after 24 h of drug treatment (Figure 1a,b). In contrast, when the concentration of H_2_O_2_ reached 10 mM and the cells were treated for 2 h, the viability of IPEC-J2 cells decreased to approximately 50% (Figure 1c). Therefore, this concentration of H_2_O_2_ was selected as the dose for inducing oxidative stress.

**Figure 2:**
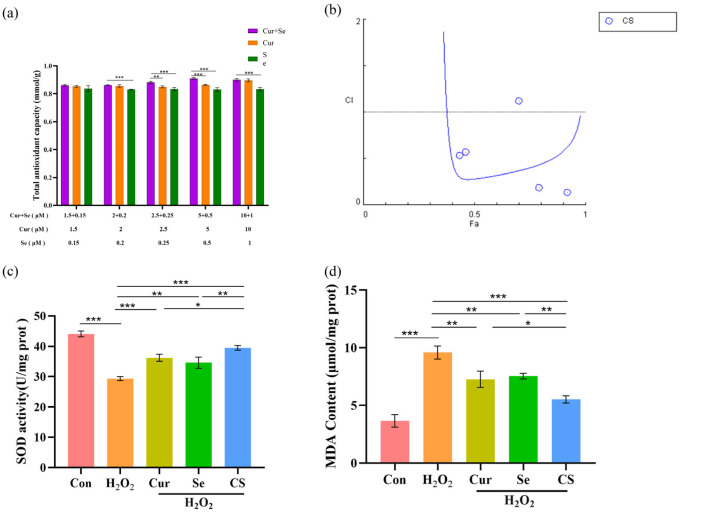

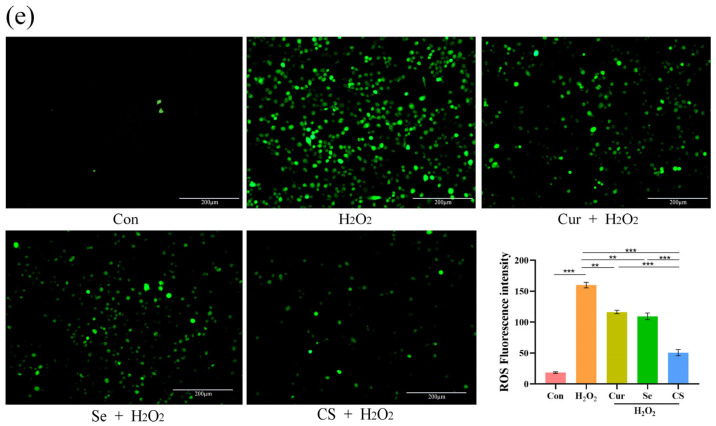
Cur and Se exert synergistic antioxidant effects in IPEC-J2 cells. (**a**) Different doses of Cur and Se synergistically mitigated the decrease in total antioxidant ability of IPEC-J2 cells induced by H_2_O_2_. (**b**) The combination index (CI) calculation revealed that Cur and Se achieved synergistic antioxidant effects, with a CI value less than 1. (**c**) Cur and Se effectively enhanced the reduction in SOD enzyme activity of IPEC-J2 cells induced by H_2_O_2_. (**d**,**e**) Cur and Se significantly attenuated the H_2_O_2_-induced elevation of MDA (**d**) and ROS (**e**) in IPEC-J2 cells. Abbreviation: Con, control. Cur, Curcumin. Se, Selenium. CS, Cur + Se. Fa, fraction affected. CI, combination index. SOD, superoxide dismutase. MDA, malondialdehyde. ROS, reactive oxygen species. The experiments were carried out in triplicate (n = 3). *, *p* < 0.05; **, *p* < 0.01; ***, *p* < 0.001.

To evaluate the synergistic antioxidant effects of Cur and Se, FRAP assays were conducted. These assays assessed the impact of different doses of Cur and Se in combination on H_2_O_2_-induced oxidative stress in IPEC-J2 cells. The results indicated that the combination of Cur and Se increased the antioxidant capacity of IPEC-J2 cells, with a superior effect compared to individual treatments (*p* < 0.05, Figure 2a). The combination index (CI) calculation revealed that Cur and Se achieved synergistic antioxidant effects, with the optimal synergy observed at a concentration of 5 μM for Cur and 0.5 μM for Se (Figure 2b, Table 3).

Moreover, the SOD, MDA, and ROS assays were conducted to confirm the synergy of Cur and Se. The results demonstrated that the MDA and ROS levels in the cells of the H_2_O_2_-treated group were significantly elevated, while the SOD activity was significantly reduced, compared to control group (*p* < 0.05); both Cur and Se, when applied individually, significantly alleviated the oxidative stress induced by H_2_O_2_ (*p* < 0.05), however, their combined application exerted more pronounced effects (Figure 2c–e).

### 3.2. Cur and Se Protect the Mitochondria of IPEC-J2 Cells Damaged by H_2_O_2_

**Figure 3:**
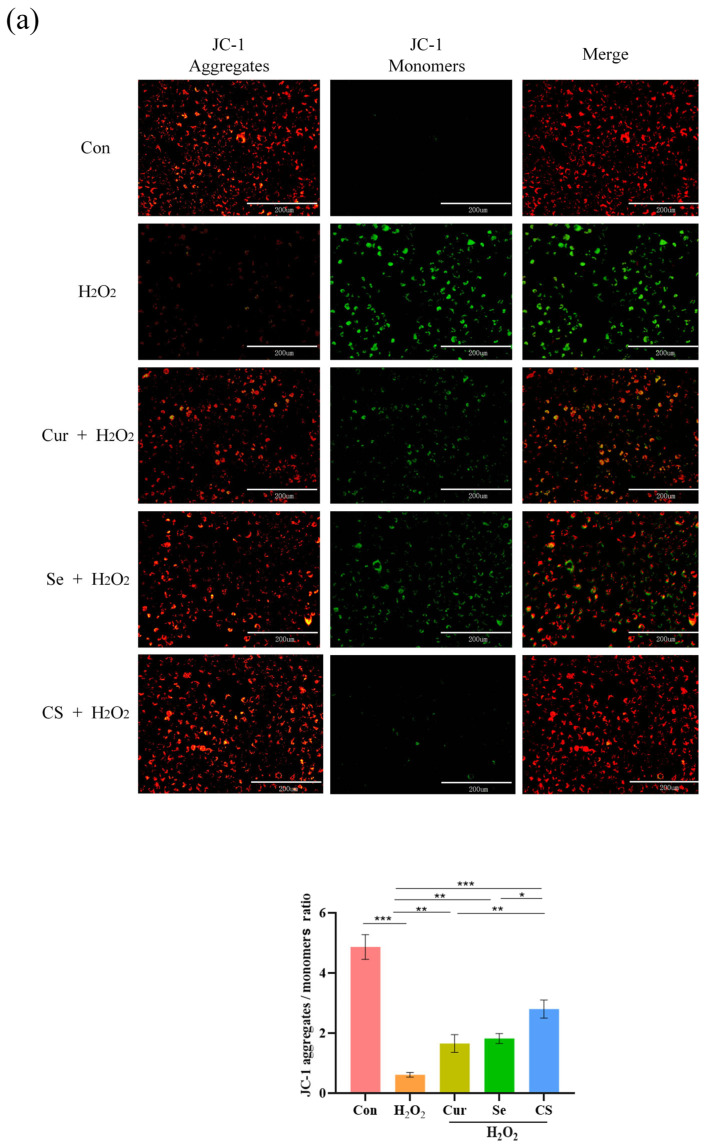

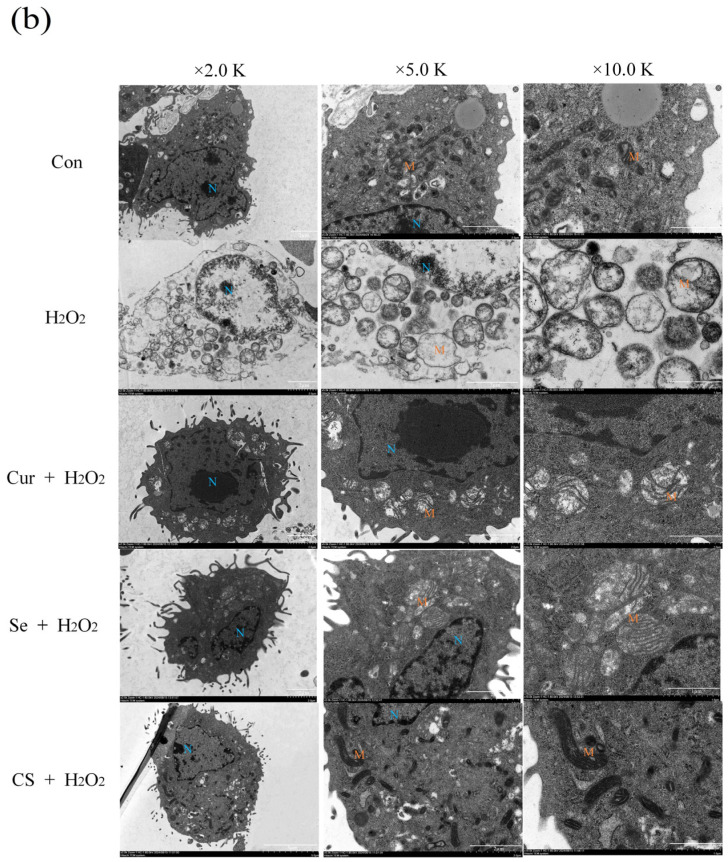
Cur and Se protect the mitochondria of IPEC-J2 cells damaged by H_2_O_2_. (**a**) The MMP of IPEC-J2 cells were detected by JC-1 probe, analyzed by fluorescence microscope. Bar: 200 μm. (**b**) Mitochondrial morphology was observed by TEM. TEM parameters: x 2.0 k/x 5.0 k/x 10.0 k, acceleration voltage 80.0 kV. Abbreviation: M, mitochondria. N, nucleus. Con, control. Cur, Curcumin. Se, Selenium. CS, Cur + Se. The experiments were carried out in triplicate, and nine TEM photographs were acquired for each group. *, *p* < 0.05; **, *p* < 0.01; ***, *p* < 0.001.

Mitochondrial membrane potential (MMP) is a key indicator of cellular energy metabolism, and its decline signifies the loss of mitochondrial function. The results of MMP detection revealed that H_2_O_2_ treatment significantly reduced MMP in IPEC-J2 cells compared to the control group (*p* < 0.05). Compared to the H_2_O_2_ group, Cur or Se alone significantly attenuated the decrease in MMP (*p* < 0.05), however, the combination of Cur and Se demonstrated a superior protective effect on MMP compared to either single treatment (*p* < 0.05, Figure 3a).

Moreover, the transmission electron microscopy (TEM) scanning results revealed that cells in the H_2_O_2_ group exhibited a large number of vacuoles, with mitochondria showing swelling, vacuolization, and disappearance of cristae, indicating that H_2_O_2_ caused severe damage to the mitochondria. In the single treatment groups with Cur or Se, the mitochondria still displayed vacuolization and swelling, but to a lesser extent than those in the H_2_O_2_ group. The mitochondrial morphology in the combination group was closest to that of the control group (Figure 3b).

### 3.3. Cur and Se Alleviate DSS-Induced Colitis in ICR Mice

To evaluate the in vivo antioxidant potential of Cur and Se, a colitis mice model was made, and the results showed that the length of the colon in DSS-treated mice was significantly shortened compared with the control group, and the mice body weight was also significantly decreased (*p* < 0.05). In contrast, the colonic length and body weight of mice in the Cur and Se monotherapy groups, as well as in the different combination dose groups, were improved significantly compared to the DSS group (Table 4).

In addition, the DAI scores were calculated based the weight loss rate, fecal consistency, and presence of hematochezia (bloody stool) of the mice. The results indicated that the DAI score of the DSS group was significantly higher than that of the control group (*p* < 0.01). Compared with the DSS group, administrations with Cur, Se, or their combination all significantly reduced the DAI in mice, with the lowest DAI observed in the M-CS group (Table 4). However, no significant differences were found between the M-CS group and the other drug-treated groups (*p* > 0.05).

### 3.4. Cur and Se Ameliorate DSS-Induced Colonic Morphology and Barrier Damage in ICR Mice

**Figure 4:**
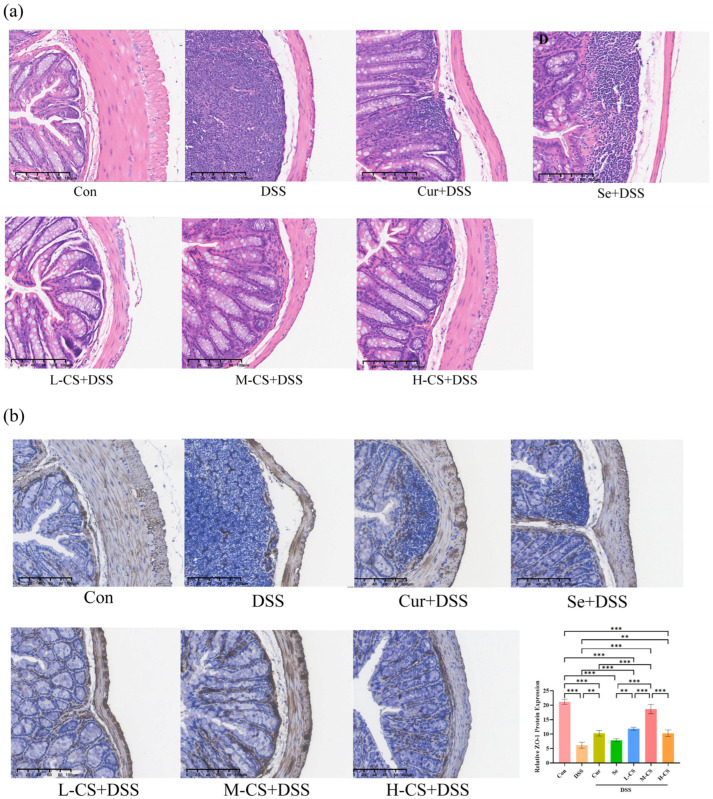
Cur and Se ameliorate DSS-induced colonic morphology and barrier damage in ICR mice. (**a**) The H&E staining of the colonic tissues of ICR mice. Bar: 100 μm. (**b**) The expression of ZO-1 protein was assessed using IHC assays. Bar: 100 μm. Abbreviation: Con, control. DSS, dextran sulfate sodium. Cur, Curcumin. Se, Selenium. L-CS, low-dose Cur + Se. M-CS, medium-dose Cur + Se. H-CS, High-dose Cur + Se. ZO-1, Zonula Occludens-1. Six photographs were taken for each group. **, *p* < 0.01; ***, *p* < 0.001.

The H&E staining results demonstrated that, in the control group, the colonic tissue exhibited a clear and intact colonic wall structure, with an intact epithelium, evenly distributed and regularly arranged glands, normal crypts, and abundant goblet cells. Additionally, there was no obvious congestion or edema, and no inflammatory cell infiltration was observed. In comparison with the control group, the colonic wall of the DSS model group was thickened and structurally disorganized, characterized by epithelial detachment, destruction, or even disappearance of glands and crypts, absence of goblet cells, and pronounced infiltration of inflammatory cells in the mucosa and submucosa. Both Cur and Se partially alleviated DSS-induced colonic morphological damage, while the colonic morphology of mice in the combination groups was closer to that of the control group (Figure 4a).

To assess the effects of Cur combined with Se on intestinal barrier function in mice, the expression of the tight junction protein ZO-1 was assessed using IHC assays. The ZO-1 protein was positively expressed in the colonic tissue of control group mice. In comparison with the control group, the expression of ZO-1 protein in the colonic tissue of the DSS group was significantly downregulated (*p* < 0.01). Compared with the DSS group, the difference in the Se group was not significant (*p* > 0.05), while the Cur group and the combination group showed significant increases (*p* < 0.05). Moreover, the expression level of ZO-1 protein in the colonic tissue of mice in the M-CS group was higher than that in the other drug-treated groups (*p* < 0.05, Figure 4b).

### 3.5. Cur and Se Alleviate DSS-Induced Oxidative Stress in ICR Mice

**Figure 5:**
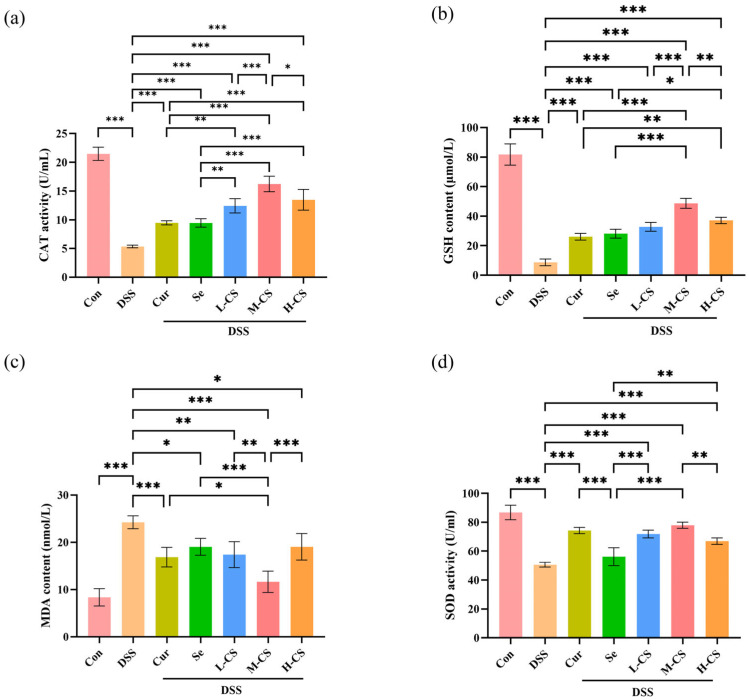
Cur and Se alleviate DSS-induced oxidative stress in ICR mice. (**a**–**d**) The activity and content of CAT, GSH, MDA, and SOD in the serum of mice were detected. (**a**) Cur and Se effectively enhanced the reduction in CAT of ICR mice serum induced by DSS. (**b**) Cur and Se effectively enhanced the reduction in GSH of ICR mice serum induced by DSS. (**c**) Cur and Se significantly attenuated the DSS-induced elevation of MDA in ICR mice serum. (**d**) Cur and Se effectively enhanced the reduction in SOD enzyme activity of ICR mice serum induced by DSS. Abbreviation: Con, control. DSS, dextran sulfate sodium. Cur, Curcumin. Se, Selenium. L-CS, low-dose Cur + Se. M-CS, medium-dose Cur + Se. H-CS, High-dose Cur + Se. CAT, catalase. GSH, glutathione. MDA, malondialdehyde. SOD, superoxide dismutase. The experiment was repeated six times (n = 6). *, *p* < 0.05; **, *p* < 0.01; ***, *p* < 0.001.

To evaluate the antioxidant capability of Cur and Se in ICR mice, the activity and content of CAT, GSH, MDA, and SOD in the serum of mice were detected. In comparison with the control group, the DSS group showed a marked reduction in CAT activity, SOD activity, and GSH content, while the MDA level was significantly elevated (*p* < 0.01); compared with the DSS group, Cur and Se both demonstrated significant antioxidant effects (*p* < 0.05), however, the serum antioxidant enzyme indices in the combination group were superior, particularly in the M-CS group (Figure 5).

### 3.6. Cur and Se Promote Antioxidant and Anti-Inflammatory Gene Expression in Mice Colon Tissues

**Figure 6:**
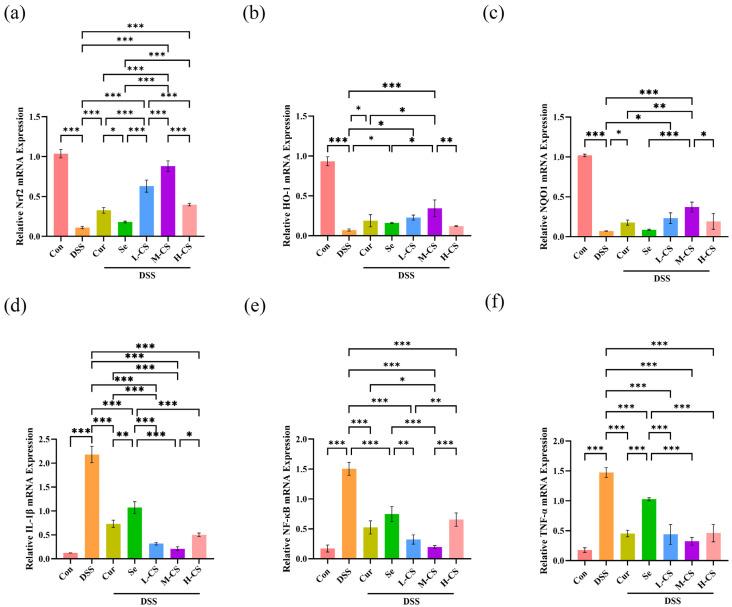
Cur and Se promote antioxidant and anti-inflammatory genes expression in mice colon tissues. (**a**–**f**) The mRNA expression levels of the Nrf2 and NF-κB pathway-related genes in mice colonic tissues were determined by RT-qPCR. (**a**) Cur and Se mitigated the downregulation of *Nrf2* gene expression in the colonic tissue of ICR mice induced by DSS. (**b**) Cur and Se reversed the downregulation of *HO-1* gene expression in the colonic tissue of ICR mice induced by DSS. (**c**) Cur and Se attenuated the downregulation of *NQO-1* gene expression in the colonic tissue of ICR mice induced by DSS. (**d**) Cur and Se decreased the elevated *IL-1β* gene expression in the colonic tissue of ICR mice induced by DSS. (**e**) Cur and Se reversed the upregulated *NF-κB* gene expression in the colonic tissue of ICR mice induced by DSS. (**f**) Cur and Se reversed the upregulated *TNF-α* gene expression in the colonic tissue of ICR mice induced by DSS. Abbreviation: Con, control. DSS, dextran sulfate sodium. Cur, Curcumin. Se, Selenium. L-CS, low-dose Cur + Se. M-CS, medium-dose Cur + Se. H-CS, High-dose Cur + Se. The experiment was repeated six times (n = 6). *, *p* < 0.05; **, *p* < 0.01; ***, *p* < 0.001.

The Nrf2/HO-1/NQO-1 signaling pathway and the NF-κB signaling pathway are closely related to antioxidant and anti-inflammatory functions. To explore the potential antioxidant signaling mechanisms of Cur and Se, the mRNA expression levels of relevant genes in mice colonic tissues were determined by RT-qPCR. In comparison with the control group, the colonic tissues of the DSS group showed a significant decrease in the mRNA expression of *Nrf2*, *HO-1*, and *NQO-1*, whereas the mRNA expression of *IL-1β*, *NF-κB*, and *TNF-α* was markedly elevated (*p* < 0.05). Compared with the DSS group, both Cur and Se alone significantly upregulated the expression of the *Nrf2* gene and downregulated the expression of *IL-1β*, *NF-κB*, and *TNF-α* genes (*p* < 0.05) but had no significant effect on the expression of *HO-1* and *NQO-1* genes (*p* > 0.05). In contrast, compared with the DSS and monotherapy groups, the colonic tissues of mice in the M-CS group exhibited significant upregulation of *Nrf2*, *HO-1*, and *NQO-1* gene expression and significant downregulation of *IL-1β*, *NF-κB*, and *TNF-α* gene expression (*p* < 0.05). These findings indicated that the synergistic antioxidant effects of Cur and Se might be associated with the activation of the Nrf2/HO-1/NQO-1 pathway and the inhibition of the NF-κB-related pathway in colonic tissues of mice (Figure 6).

### 3.7. Cur and Se Modulate the Gut Microbiota Dysbiosis Induced by DSS in ICR Mice

**Figure 7:**
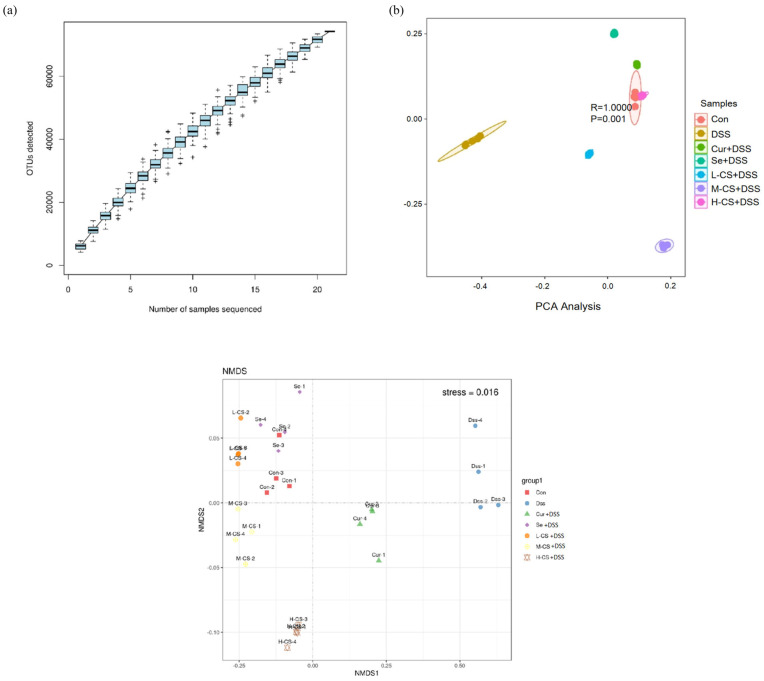

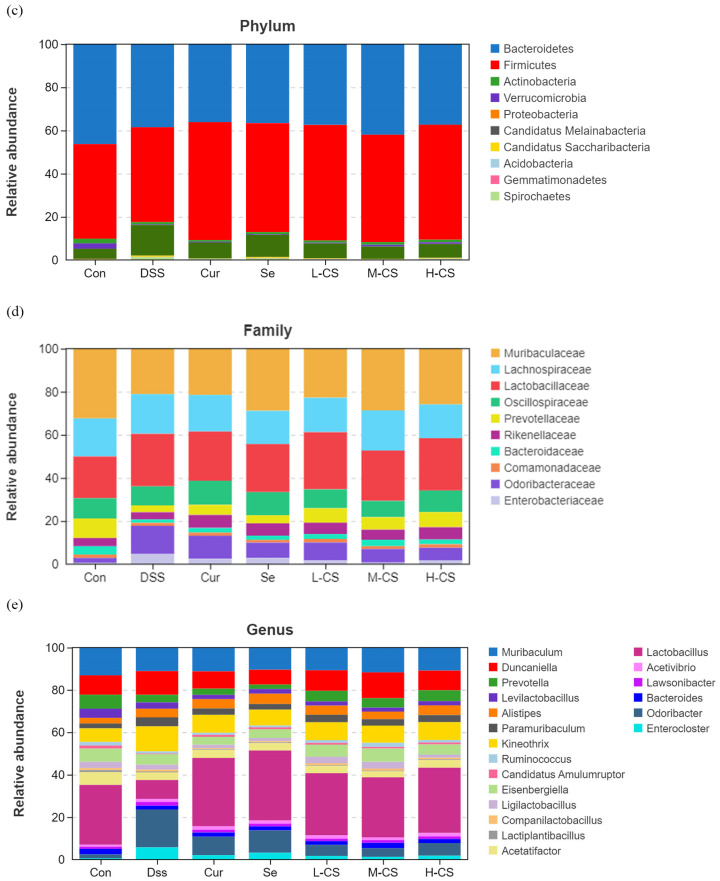
Cur and Se modulate the gut microbiota dysbiosis induced by DSS in ICR mice. (**a**) The petal plot and the species accumulation curve of 16S rRNA transcriptome sequencing of intestinal microbiota in ICR mice. (**b**) Principal components analysis (PCA) and non-metric multidimensional scaling (NMDS) analysis of 16S rRNA transcriptome sequencing of intestinal microbiota in ICR mice. (**c**) Stacked plot of intestinal microbiota at phylum level in ICR mice. (**d**) Stacked plot of intestinal microbiota at family level in ICR mice. (**e**) Stacked plot of intestinal microbiota at genus level in ICR mice. Abbreviation: Con, control. DSS, dextran sulfate sodium. Cur, Curcumin. Se, Selenium. L-CS, low-dose Cur + Se. M-CS, medium-dose Cur + Se. H-CS, High-dose Cur + Se. The experiment was conducted in quadruplicate (n = 4). The data are expressed as the mean ± SD.

The gut microbiota is crucial for intestinal health. In order to evaluate the effects of Cur and Se on the gut microbiota, 16S rRNA sequencing was performed on fecal samples from the mice. A total of 785 OTUs were shared among the seven experimental groups, and the sequencing quantity and depth were sufficient (Figure 7a).

The results of alpha diversity analysis indicated that DSS treatment led to a reduction in the richness and diversity of the gut microbiota in mice (*p* < 0.05). Compared with the DSS and monotherapy groups, Cur combined with Se significantly increased the diversity of the colonic microbial community and improved the richness of the microbiota (*p* < 0.05, Table 5).

The beta diversity analysis revealed that the gut microbiota of mice in the DSS group was distinctly separated from that of the control group. The gut microbial structure of mice treated with Cur and Se, either individually or in combination, showed a noticeable recovery. Among them, the microbiota profile of the combination treatment group more closely resembled that of the control group (Figure 7b).

At the phylum level, the abundance of *Bacteroidetes* and *Firmicutes* in the gut of mice in the DSS group was significantly reduced, while the abundance of *Proteobacteria* was increased compared with the control group (*p* < 0.05). Both Cur and Se significantly increased the abundance of *Bacteroidetes* and *Firmicutes* and decreased the abundance of *Proteobacteria*, with the combination treatment group showing superior effects compared to the monotherapy groups (*p* < 0.05, Figure 7c).

At the family level, in comparison with the control group, the abundance of *Muribaculaceae*, *Prevotellaceae*, and *Comamonadaceae* in the gut of mice in the DSS group was significantly decreased (*p* < 0.05). Both Cur and Se significantly increased the abundance of these bacterial families, and the combined treatment group exhibited a more pronounced effect than either monotherapy group (*p* < 0.05, Figure 7d).

At the genus level, in comparison with the control group, the abundance of *Muribaculum*, *Levilactobacillus*, and *Ruminococcus* in the gut of mice in the DSS group was significantly reduced, while the abundance of *Odoribacter* was significantly increased (*p* < 0.05). Both Cur and Se significantly reversed the effects of DSS, with the combined treatment group showing superior efficacy compared to the monotherapy groups (*p* < 0.05, Figure 7e).

## 4. Discussion

Oxidative stress is associated with a variety of diseases in pigs, especially diarrhea [3]. Excessive ROS can disrupt the integrity of the lipid bilayer of the cell membrane and alter enzyme activity, receptor function, and signaling pathway transmission [39,40]. Simultaneously, ROS induce DNA damage and cause protein oxidation, resulting in amino acid cross-linking, enzyme inactivation, and metabolic disturbance, resulting in cell death [41]. Various antioxidant strategies have been proposed, including nutritional methods [42], non-invasive continuous monitoring [43], and breeding [44]. Cur combined with Se has shown great potential in regulating oxidative stress in poultry [29]. Cur possesses strong antioxidant capacity and is effective in alleviating oxidative damage in the pig intestinal tract, however, its clinical application is limited by low solubility, stability, and bioavailability [45,46,47]. The safe dosage range of Se is relatively narrow, and excessive Se content in feed can lead to poisoning symptoms in pigs, including growth inhibition, hair loss, and liver or kidney damage [48]. Since the combined use of drugs may enhance efficacy or reduce toxicity through synergistic interactions, the co-application of Cur and Se could potentially compensate for their individual limitations.

In the current study, multiple doses of Cur or Se were applied both in vitro and in vivo. Different dose combinations of Cur and Se exhibited distinct effects. For instance, in the in vitro experiments, the optimal CI value appeared at Cur 5 μM and Se 0.5 μM (Table 3), rather than at the higher doses of Cur 10 μM and Se 1 μM. Similarly, in the in vivo experiments, the best antioxidant effect was observed with medium Cur combined with Se. This is because the interaction between drugs, represented by the combination index (CI) value, can exhibit a curved pattern [49], where the optimal drug combination may not necessarily be the one with the highest doses. This phenomenon has also been observed in other studies involving drug combinations [29,50,51]. This finding underscores the necessity of conducting dose-gradient experiments in combination therapy to identify the optimal dosing regimen.

In this study, IPEC-J2 cells were employed for in vitro experiments, while ICR mice served as the in vivo model. Although several previous studies have similarly utilized mice as a model for pigs [52,53,54], it is acknowledged that significant differences exist between the in vivo environments of mice and pigs. Therefore, it is essential for future research to conduct trials in experimental pigs to verify the antioxidant effects of Cur and Se, determine their appropriate dosages, and assess their safety in pigs.

The Nrf2/HO-1/NQO-1 pathway is critical in antioxidative stress [55], it has been confirmed in studies related to pigs that they are involved in various physiological and pathological processes [56]. Under regular physiological conditions, Nrf2 exists in the cytoplasm in combination with kelch-like ECH-associated protein 1 (Keap1), and when subjected to oxidative stress, Nrf2 dissociates from the Keap1 complex and transfers to the nucleus, binds to the antioxidant response elements, and initiates the transcription and expression of antioxidant genes, such as *HO-1*, *NQO-1*, etc. [57]. In models of oxidative stress [58], intestinal injury [35], and infection disease in pigs [56], activation of this pathway significantly alleviates oxidative damage, highlighting its value as an important antioxidant intervention target. In addition, the Nrf2/HO-1/NQO-1 pathway also has cross-regulation with the NF-κB inflammatory pathway or mitogen-activated protein kinase (MAPK) pathway [59,60,61]. Activation of Nrf2 can inhibit the release of pro-inflammatory factors mediated by NF-κB, achieving a synergistic antioxidant and anti-inflammatory effect [62]. The results of present study indicated that Cur and Se significantly activated the Nrf2 signaling pathway and inhibit the NF-κB signaling pathway in mouse colonic tissues. However, further research is needed to clarify the relationship between the combination therapy and these two signaling pathways. For example, Western blot assays could be used to detect the expression and modification of related proteins, and immunofluorescence assays could be employed to examine the nuclear translocation of key proteins. Additionally, introducing knockdown or overexpression of key genes before assessing the antioxidant effects of Cur and Se could offer deeper insights into their antioxidant mechanisms.

Mitochondrial dynamics, particularly mitochondrial fusion, is vital for mitochondrial function and has been implicated in various diseases [63,64]. Fusion enhances mitochondrial activity by facilitating the exchange of contents between mitochondria [63]. In this study, the TEM results showed that the mitochondrial length of cells in the combination group appeared longer than that of cells in the other groups (Figure 3b), indicating that mitochondrial fusion might have occurred in response to Cur and Se treatment. Additionally, both Cur and Se have been reported to improve mitochondrial dynamics individually [65,66]. This fusion could potentially be another mechanism underlying the combined antioxidant actions of Cur and Se. However, to confirm this observation, future studies should investigate the expression of proteins associated with mitochondrial fusion, such as Mitofusin 1/2 and Dynamin related protein 1 (DRP1).

Intestinal oxidative stress disrupts the intestinal barrier function, causing mitochondrial dysfunction [67] and intestinal flora imbalance [68], which ultimately leads to the occurrence and development of intestinal inflammation [69]. The imbalance of gut microbiota structure is associated with the onset and progression of various diseases, especially in pig diarrhea [70]. The normal gut microbiota maintains intestinal barrier integrity and regulates immune function via metabolic products such as short-chain fatty acids. In contrast, dysbiosis of the microbiota can increase oxidative stress and inflammatory responses in the gut, thereby inducing diarrhea [71,72,73]. The results of the present study showed that Cur combined with Se significantly improved the intestinal barrier in ICR mice with colitis and reversed DSS-induced gut microbiota dysbiosis. For instance, the phylum *Firmicutes* is essential for maintaining gut homeostasis [74,75], and it has been reported that DSS can induce a reduction in the abundance of *Firmicutes* within the intestinal microbiota of mice [76,77], which is consistent with the results of the present study. Compared with the DSS group, Cur combined with Se could significantly increase the abundance of *Firmicutes* in the intestinal microbiota of ICR mice (Figure 7), which is meaningful for maintaining gut microbiota homeostasis and promoting gut health. However, there are differences in the gut microbiota between mice and pigs. In the future, it is necessary to further elucidate the effects of the combination therapy on the pig gut microbiota and to investigate the relationship between the changes in the microbiota and the host’s antioxidant functions.

## 5. Conclusions

The combination of Cur and Se exerted synergistic antioxidant effects on H_2_O_2_-induced oxidative damage in IPEC-J2 cells. Furthermore, this combination alleviated DSS-induced colitis in mice, protected the colonic tissue structure, and promoted the restoration of intestinal flora homeostasis. Meanwhile, these effects are related to the Nrf2/HO-1/NQO-1 and NF-κB signaling pathways. The results are expected to facilitate the future application of Cur and Se in antioxidant strategies for swine feeding.

## Figures and Tables

**Table 1:** Groups for experimental animals.

Group	1–14 Day Processing	15–21 Day Processing
Blank control group (Con)	Normal feed	Purified water for drinking
Model group (DSS)	Normal feed	Drinking a 3% solution of DSS
Curcumin group (Cur)	Gastric curcumin 200 mg/kg	Drinking a 3% solution of DSS
Sodium selenite group (Se)	Sodium selenite 2 mg/kg	Drinking a 3% solution of DSS
Low-dose curcumin combined with sodium selenite group (L-CS)	Gastric curcumin 50 mg/kg and sodium selenite 2 mg/kg	Drinking a 3% solution of DSS
Medium-dose curcumin combined with sodium selenite group (M-CS)	Gastric curcumin 100 mg/kg and sodium selenite 2 mg/kg	Drinking a 3% solution of DSS
High-dose curcumin combined with sodium selenite group (H-CS)	Gastric curcumin 200 mg/kg and sodium selenite 2 mg/kg	Drinking a 3% solution of DSS

**Table 2:** RT-qPCR primer sequence information.

Genes	Primer Sequences	Product Length
*Nrf*2 (mus)	F: ACACGAGATGAGCTTAGGGC R: TCGGATCAATGCGAGCTGAG	135
*HO*-1 (mus)	F: CAGAAGAGGCTAAGACCGCC R: CTCTGACGAAGTGACGCCAT	118
*NQO*-1 (mus)	F: GCGAGAAGAGCCCTGATTGT R: TTCGAGTCCTTCAGCTCACC	185
*IL*-1*β* (mus)	F: GAAATGCCACCTTTTGACAGTG R: TGGATGCTCTCATCAGGACAG	116
*TNF-α* (mus)	F: GCCGATGGGTTGTACCTTGT	139
	R: TCTTGACGGCAGAGAGGAGG	
*NF-κB* (mus)	F: GTTTGATGCTGATGAAGACTTGG	186
	R: GTCACCAGGCGAGTTATAGC	
*β-actin* (mus)	F: TGTCCACCTTCCAGCAGATGT R: AGCTCAGTAACAGTCCGCCTAG	101

**Table 3:** CI Value of Cur and Se.

Cur (μM)	Se (μM)	CI Value	Combined Effect
1.5	0.15	0.5317	synergistic effect
2	0.2	0.5721	synergistic effect
2.5	0.25	0.1828	synergistic effect
5	0.5	0.1350	synergistic effect
10	1	1.1198	antagonistic effect

Abbreviation: Cur, Curcumin. Se, Selenium. Fa, fraction affected. CI, combination index.

**Table 4:** Effects of Cur and Se on colon length, weight, and final DAI score in DSS-induced colitis in ICR mice.

Group	Length of the Colon (cm)	Weight (g)	Last DAI Score
Con group	8.68 ± 0.52 ^a^	25.47 ± 0.51 ^a^	0.00 ^c^
DSS group	5.27 ± 0.56 ^c^	22.10 ± 0.48 ^d^	2.61 ± 0.26 ^a^
Cur group	7.42 ± 0.50 ^b^	24.15 ± 0.51 ^bc^	1.46 ± 0.13 ^b^
Se group	7.18 ± 0.44 ^b^	24.15 ± 0.46 ^bc^	1.60 ± 0.21 ^b^
L-CS group	7.52 ± 0.28 ^a^	25.40 ± 0.49 ^b^	1.34 ± 0.11 ^b^
M-CS group	7.55 ± 0.29 ^a^	25.27 ± 0.51 ^b^	1.25 ± 0.22 ^b^
H-CS group	6.65 ± 0.33 ^b^	24.15 ± 0.61 ^c^	1.44 ± 0.31 ^b^

Note: The same letter (a, b, c, d, e) in the shoulder labels indicates no significant difference (*p* > 0.05), while different letters represent significant differences (*p* < 0.05). The data are expressed as the mean ± SD (n = 10).

**Table 5:** Alpha diversity analysis of the 16srRNA gene sequencing.

Group	Chao1	ACE	Shannon	Simpson
Con	9478.71 ± 222.54 ^a^	11,662.89 ± 553.83 ^a^	7.76 ± 0.03 ^a^	0.97
Dss	4657.31 ± 286.67 ^d^	5763.14 ± 270.94 ^e^	6.44 ± 0.34 ^c^	0.99
Cur	6171.22 ± 135.12 ^c^	7475.53 ± 184.67 ^cd^	6.76 ± 0.60 ^abc^	0.98
Se	6018.91 ± 106.63 ^c^	6273.96 ± 152.24 ^de^	6.62 ± 0.75 ^bc^	0.98
L-CS	6945.48 ± 171.19 ^b^	7984.11 ± 132.98 ^bc^	7.09 ± 0.18 ^abc^	0.97
M-CS	7341.19 ± 156.85 ^b^	8915.53 ± 226.58 ^b^	7.61 ± 0.07 ^ab^	0.97
H-CS	6180.65 ± 137.24 ^c^	7225.53 ± 240.11 ^cd^	7.45 ± 0.20 ^abc^	0.98

Note: The same letter (a, b, c, d) in the shoulder labels indicates no significant difference (*p* > 0.05), while different letters represent significant differences (*p* < 0.05). The experiment was conducted in quadruplicate (n = 4). The data are expressed as the mean ± SD.

## Data Availability

Data will be made available on request. The raw 16s rRNA sequencing data have been uploaded to the NCBI SRA database (accession number: PRJNA1291475).

## Supplementary Materials

The following supporting information can be downloaded at: https://www.mdpi.com/article/10.3390/biology14091117/s1, Table S1: Disease activity index criteria.
