# Supplementary material for: Curcumin and Selenium Synergistically Alleviate Oxidative Stress in IPEC-J2 Cells and ICR Mice
Source: Biology (Basel). 2025 Aug 23;14(9):1117. doi: 10.3390/biology14091117 (PMC12467055; doi:10.3390/biology14091117)
Supplement: Supplementary file 1 [file biology-14-01117-s001.zip › biology-3790378-supplementary.pdf]

Supplemental Table S1. Disease activity index criteria.

Scoring	Rate of weight loss（%）	Stool consistency	The situation of hematochezia
0	none	normal	occult blood test（-）
1	1-5	soft but molded	occult blood test（+）
2	6-10	unformed stool	have blood in one's stool
3	11-15	unformed stool	have blood in one's stool
4	＞15	watery stool	bloody stools in the flesh
